# Accuracy of CREST Guideline in Management of Cellulitis in Emergency Department; a Systematic Review and Meta-analysis

**DOI:** 10.22037/aaem.v9i1.1422

**Published:** 2021-11-03

**Authors:** Hossein Akhavan, Seyed Reza Habibzadeh, Fatemeh Maleki, Mahdi Foroughian, Sayyed Reza Ahmadi, Reza Akhavan, Bita Abbasi, Behzad Shahi, Navid Kalani, Naser Hatami, Amir Mangouri, Sheida Jamalnia

**Affiliations:** 1 Department of Pediatrics, Faculty of Medicine, Mashhad University of Medical Sciences, Mashhad, Iran.; 2 Department of Emergency Medicine, Faculty of Medicine, Mashhad University of Medical Sciences, Mashhad, Iran.; 3 Department of Emergency Medicine, Faculty of Medicine, Birjand University of Medical Sciences, Birjand, Iran.; 4 Department of Radiology, Faculty of Medicine, Mashhad University of Medical sciences, Mashhad, Iran.; 5 Department of Emergency Medicine, Faculty of Medicine, Zahedan University of Medical Sciences, Zahedan, Iran.; 6 Research Center for Social Determinants of Health, Jahrom University of Medical Sciences, Jahrom, Iran.; 7 Student Research Committee, Jahrom University of Medical Sciences, Jahrom, Iran.; 8 Division of Vascular Surgery and Endovascular Therapy, Department of General Surgery, Sina Hospital, Tehran University of Medical Sciences, Tehran, Iran.; 9 Medical Journalism Department, Shiraz University of Medical Sciences, Shiraz, Iran.

**Keywords:** Cellulitis, emergency service, hospital, systematic review, skin diseases, bacterial, anti-bacterial agents

## Abstract

**Introduction:**

Skin and soft tissue infections are important causes of outpatient visits to medical clinics or hospitals. This study aimed to review the literature for the accuracy of Clinical Resource Efficiency Support Team (CREST) guideline in management of cellulitis in emergency department.

**Method:**

Studies that had evaluated cellulitis patients using the CREST guideline were quarried in Scopus, Web of Science, and PubMed database, from 2005 to the end of 2020. The quality of the studies was evaluated using Scottish Intercollegiate Guideline Network (SIGN) checklist for cohort studies. Pooled area under the receiver operating characteristic curve (AUROC) of CREST guideline regarding the rate of hospital stay more than 24 hours, rate of revisit, and appropriateness of antimicrobial treatment in management of cellulitis in emergency department was evaluated.

**Results:**

Seven studies evaluating a total of 1640 adult cellulitis patients were finally entered to the study. In evaluation of the rate of the appropriate treatment versus over-treatment, the pooled AUROC was estimated to be 0.38 (95% confidence interval (CI): 0.06 – 0.82), indicating low accuracy (AUROC lower than 0.5) of guideline for antimicrobial choice. CREST II patients had a significantly lower odds ratio (OR) of revisiting the Emergency Department, OR=0.21 (95% CI: 0.009‎ – ‎ 0.47). Pooled AUROC value of 0.86 (CI95%: 0.84 – 0.89) showed accuracy of the CREST classification in prediction of being hospitalized more or less than 24 hours.

**Conclusion:**

CREST classification shows good accuracy in determining the duration of hospitalization or observation in ED but it could lead to inevitable over/under treatment with empirical antimicrobial agents.

## 1. Introduction

Skin and soft tissue infections are important causes of outpatient visits to medical clinics or hospitals. These infections have a wide range of symptomatology and etiology that could even be life threatening in some cases (1). Cellulitis is an acute infection of the skin and soft tissues. Subcutaneous tissues show redness, pain, and swelling in the affected area. The most common etiology is Staphylococcus aureus bacteria, followed by Streptococcus pyogenes and many other gram-positive cocci and rarely some gram-negative germs (2). Clinical evaluation of the severity of the infection is very important and decisive; however, the need for different diagnostic and therapeutic algorithms to guide physicians in reaching the right and appropriate decision has not been fully addressed (3). Koerner et al. (2011) reviewed the recent attempts in classification of cellulitis cases and stated that primary classifications were assorting cases based on the site of the infection; while further studies suggested more comprehensive guidelines as well as the Eron criteria (4). Later, Clinical Resource Efficiency Support Team (CREST) was developed, based on the Eron recommendations, addressed as Eron/CREST classification by some authors (5), with an easy method of classification for clinical application (6). But since systemic sepsis has not been fully considered in this guideline, some researchers have doubted its application in clinical practice (4). Since no study has pooled the clinical outcomes of the CREST application, this study aimed to review the literature on the accuracy of CREST guideline in management of cellulitis in emergency department. 

## 2. Methods

***2.1. Study design and setting***

This study was performed in adherence to the guidelines of the Preferential Cases of The Report for Systematic Review and Meta-Analysis (PRISMA) and is a systematic review and meta-analysis of the existing literature on the accuracy of CREST guideline in treatment of cellulitis, published in peer-reviewed journals. English language studies were quarried among all articles published from 2005 to the end of 2020, on the topic of cellulitis, since CREST guideline was first established in 2005. Studies which evaluated their study population using the CREST guideline were searched in Scopus, Web of Science, and PubMed databases by two researchers using the keywords of "CREST ", "cellulitis" and "bacterial skin infection". Only articles about cellulitis that contained the keywords of CREST were included in initial search. 

***2.2. Search strategy***

In PubMed, using the search strategy of [(CREST‎ ‎OR Eron) and (Cellulitis OR bacterial skin infection)], 44 results were found. Along with multiple Scopus and Web of Science search results found using the same strategy, in the initial search, 135 potentially relevant articles were retrieved, 127 of which remained after removing the duplicate items. Then, articles were selected based on the title and abstract, a list of abstracts was prepared. After hiding the details of the articles such as the author's name, the name of the journal, etc., the full texts of the articles were given to 2 trained researchers to review the text of articles. Each article was reviewed by 2 independent researchers and in case of rejection of the articles by both researchers, the reason was mentioned and in case of disagreement between them, the article was judged by a third person. 115 articles were excluded from the study due to irrelevance or not containing CREST guideline in their methodology. Finally, 12 articles met the inclusion criteria and entered the quality assessment process. 

***2.3. Quality assessment***

A checklist of Scottish intercollegiate guidelines network (SIGN) was used to evaluate the quality of these articles (7). Seven articles had acceptable quality for being included in study. 

***2.4. Statistical analysis***

A web-based calculator was used to determine Area under the Receiver operating characteristic (ROC) Curve (AUROC) of the overall accuracy of CREST in predicting the rate of hospital stay more than 24 hours (8). To combine the event rates or values of AUROC with respect to the percentage or standard error of the values, the weighted average and the random effects were used in the meta-analysis due to the heterogeneity of the studies. The I^2^ index and the Cochran test were used to examine the heterogeneity between the results. The Egger’s test and funnel plot were used to examine the publication bias in the Revman software version 5.4.1.

In this meta-analysis, AUROC of CREST in predicting the studied variables was estimated and entered in a random effects model, due to high heterogeneity (I^2^=99%). 

## 3. Results

**3.1. Characteristics of included studies**

**Figure 1:**
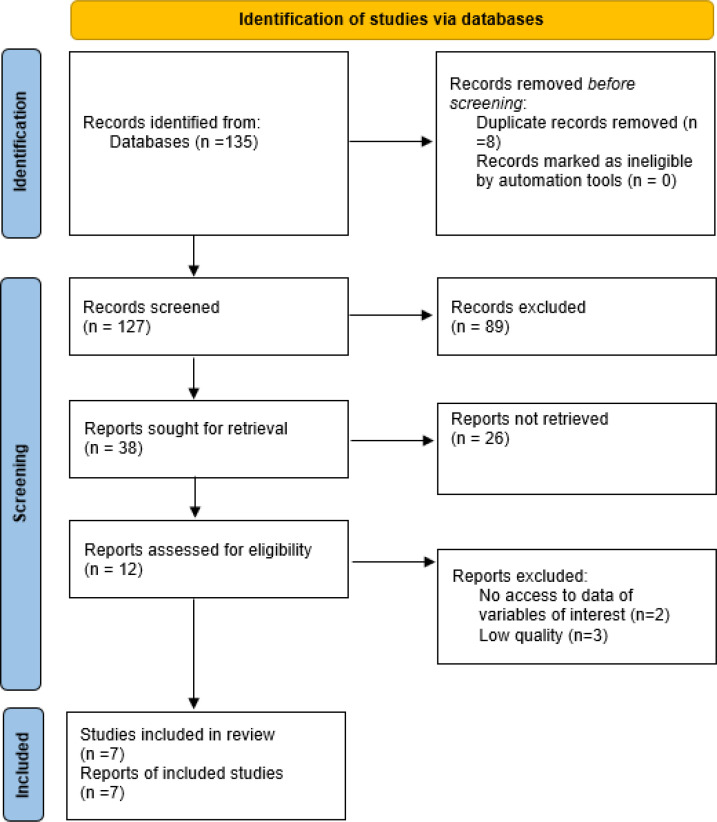
PRISMA flow chart of study

Seven studies were finally entered into the quantitative synthesis ‎in this meta-analysis, as shown in figure 1. Six studies were prospective ‎cohort studies and one was retrospective. Two studies were conducted in Iran, 3 studies in USA, and 2 in Scotland. Study ‎setting was Emergency department (ED) in 4 studies; while 3 of the studies evaluated hospitalized patients (9-11). A total of 1640 patients were studied in our meta-analysis. All studies evaluated adult subjects (over 18 years old). We were not able to classify studies into subgroups for meta-analysis to see the possible differences between old and young adults. All studies had the same inclusion criterion, which was diagnosis of cellulitis. Although evaluating distinct areas of cellulitis involvement, none of the studies included facial cellulitis. Various outcomes were measured in different studies, so we could not include all studies in‎a quantitative synthesis for a particular outcome (Table 1). 

3.2. Accuracy of CREST guideline

***For hospitalization length***

**Figure 2:**
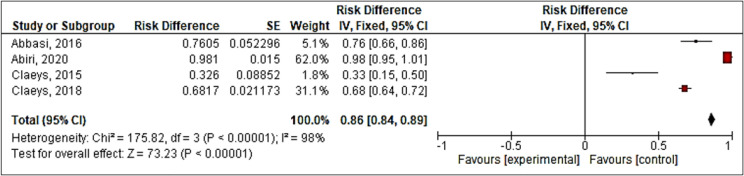
Forest plot of Clinical Resource Efficiency Support Team (CREST) guideline’s accuracy for hospitalization length; less versus more than 24 hours (based on pooled area under the Receiver Operating Characteristic (ROC) curve)

Results showed pooled AUROC value of 0.86 (95%CI: 0.84 – 0.89) for CREST guideline regarding hospitalization length based on the combination of results from 4 studies referenced in figure 2. But there was a high heterogeneity (I^2^=98%). No further subgroup analysis was possible to determine the source of heterogeneity. 

***For empirical antimicrobial choices***

**Figure 3:**
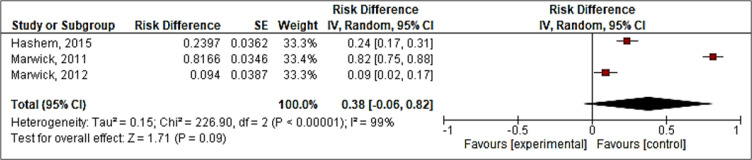
Forest plot of Clinical Resource Efficiency Support Team (CREST) guideline’s accuracy for appropriate antimicrobial treatment (based on pooled area under the Receiver Operating Characteristic (ROC) curve)

Appropriateness of empirical antimicrobial choices was assessed in 3 studies (9-11) through evaluating biological cultures, as shown in Table 3. Results were summarized as undertreatment, overtreatment, or appropriate treatment in studies. As shown in figure 3, the pooled AUROC of CREST guideline for empirical antimicrobial choices was estimated to be 0.38, (95%CI: 0.06 – 0.82).

***For revisiting after being discharged***

**Figure 4:**
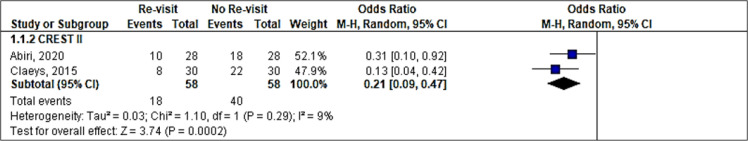
Forest plot of Clinical Resource Efficiency Support Team (CREST) guideline’s accuracy for rate of revisit after being discharged (based on pooled area under the Receiver Operating Characteristic (ROC) curve)

As shown in figure 4, revisiting after being discharged from the emergency department was evaluated in studies by Claeys (2014) and Abiri. Comparisons were only available for CREST II. CREST II patients had a significantly low odds ratio (OR) of revisiting the ED, OR=0.21 (95%CI: 0.009 – 0.47).

***3.3. Publication bias***

**Figure 5:**
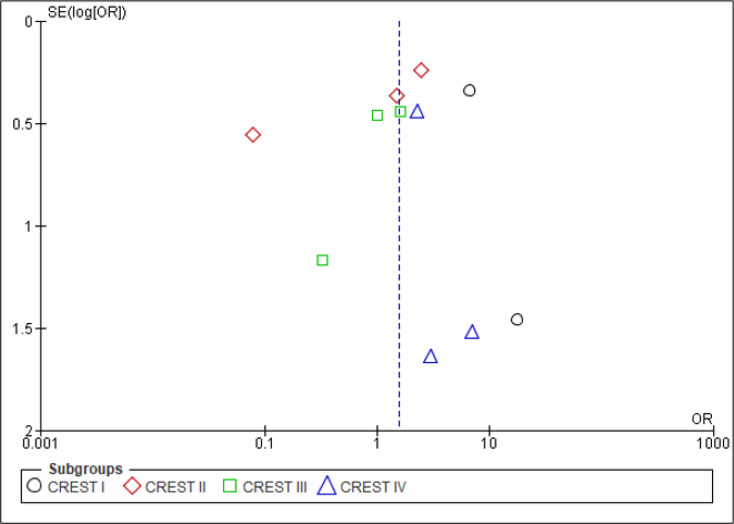
Funnel plot of the study to assess publication bias

To assess the publication bias, the funnel plot was visually inspected for asymmetry, as shown in figure 5.

## 4. Discussion

Our study revealed that the pooled AUROC for evaluating the rate of appropriate treatment versus overtreatment was 0.38 (95% CI: 0.06–0.82), indicating low accuracy (AUROC less than 0.5) of CREST guideline for antimicrobial choice. The odds of revisiting the emergency department were considerably lower in CREST II patients, with an OR of 0.21 (95%CI: 0.009–0.47). The CREST classification was shown to be accurate for being hospitalized for more than 24 hours with a pooled AUROC of 0.86 (95%CI: 0.84 – 0.89). ‎Soft tissue infections are a common group of infections that are often mild to moderate in severity and are easily treatable. Their etiological diagnosis is often difficult and unnecessary in most cases of cellulitis, where the patient has mild symptoms. Our study was a systematic review of the studies that used CREST guideline in management of cellulitis. There were few studies conducted in this area and only 7 studies were included

 in our qualitative review. Further assessment of study outcomes showed interesting findings in evaluation of empirical antimicrobial choices and hospitalization outcomes. Laboratory investigations are suggested for CREST II-IV classes and most Class I CREST classified patients get outpatient care with first line antibiotic choice of oral Flucloxacillin 500 mg per day. Patients classified in class II or higher classes may receive IV therapy (5, 6). Although pooling the data of IV versus oral treatment was not possible in our study, duration of hospital stay in ED or observation units were evaluated in some of our included studies, which revealed that patients with higher CREST classification had higher rates of staying in the hospital for more than 24 hours based on the AUROC. Staying in the hospital and observation units for more than 24 hours may also be showing the need for IV antibiotic therapy based on the findings of Claeys et al(12, 13) and Abiri et al. (14). But further laboratory investigations were not presented in all studies, as some of studies were evaluating outpatient cases, which had CREST I and II classes. 

Our study showed that the pooled AUROC for appropriateness of treatment was 0.38 (95%CI: -0.06 – 0.82), indicating no significant difference in appropriate or under/over treatment; thus, reconsiderations are needed for treatment of middle classes. The closeness of the definitions provided for classes I to IV may be the reason for uncertainty in the decisions for treatment of these cases. However, in our study, there was a large spatial heterogeneity in the included studies, which could suggest the role of differences in the pattern of antibiotic resistance of gram-positive bacteria as a heterogeneity factor. Various studies conducted in Iran show a high rate of antibiotic resistance in Iran (15, 16), the pattern of which might be different from the United States and Europe (17).

**Table 1:** Characteristics of included studies

**Study**	**Design**	**Setting**	**Outcomes **	**N**	**Inclusion criteria**
Abbasi, 2016 (18) Iran	Prospective cohort	ED	Hospitalization less or more than 24 hrevisit in 1 week	89	Non-facial cellulitis
Abiri, 2020(14) Iran	Prospective cohort	ED	Hospitalization less or more than 24 hrevisit in 1 week	100	Limb cellulitis
Hashem, 2015(11) USA	Retrospective	Hospitalized	Appropriateness of empirical antimicrobial choicesclinical response	369	Patients admitted with cellulitis
Claeys, 2015 (12) USA	Retrospective cohort	ED/or observation units	96-h ED revisit/hospitalization	308	Adult cellulitis patients with less than 24 h of IV antibiotics without hospital admission
Claeys, 2018(13) USA	Observational cohort study	ED or observation units	Area-under-the- receiver-operating-characteristic-curve (AUROC) analysis of ED/OU versus inpatient	506	Diagnosis of acute bacterial skin and skin structure infections
Marwick, 2011(9) Scotland	Retrospective cohort	Hospitalized	Appropriateness of empirical antimicrobial choices	189	Received antibiotic treatment for cellulitis in hospital
Marwick, 2012 (10) Scotland	Cohort	Acute	Appropriateness of empirical antimicrobial choices	79	Adult patients with cellulitis

NA: Not Addressed; ED: Emergency Department; OU: Outpatient.

**Table 2:** Quality of studies included in the meta-analysis based on the Scottish intercollegiate guidelines network (SIGN) checklist

Study	**Appropriate and clearly focused question**	**Predicting the outcome at the time of enrolment**	**Lost to follow up status addressed**	**Clearly defined outcomes**	**A reliable method of exposure assessment**	**Evidence of outcome assessment**	**Exposure level or prognostic factor is assessed more than once**	**The main potential confounders are identified and taken into account in the design and analysis**	**Have confidence intervals been provided?**	**Minimization of the risk of bias or confounding?**	**Clear evidence of an association between exposure and outcome?**	**Are the results of this study directly applicable to the patient group targeted in this guideline?**
Abbasi, 2016	Y	Y	NA	Y	Y	N	N	N	Y	N	Y	Y
Abiri, 2020	Y	Y	NA	Y	Y	N	N	N	N	Y	Y	Y
Hashem, 2015	Y	Y	Y	Y	Y	Y	Y	Y	N	Y	Y	Y
Claeys, 2015	Y	Y	Y	Y	Y	Y	Y	Y	Y	Y	Y	Y
Claeys, 2018	Y	Y	Y	Y	Y	Y	Y	Y	Y	Y	Y	Y
Marwick, 2011	Y	Y	Y	Y	Y	N	Y	N	Y	Y	Y	Y
Marwick, 2012	Y	Y	Y	Y	Y	Y	Y	Y	Y	Y	Y	Y

N: no; NA: not addressed; Y: yes.

**Table 3:** Quality of antimicrobial treatment based on the Clinical Resource Efficiency Support Team (CREST) guideline

	Treatment		**CREST I**	**CREST II**	**CREST III**	**CREST IV**
**Marwick, 2012**		Appropriate	0(0)	4(4.6)	1(1.15)	18(20.69)
		Under/Over	19(21.84)	33(37.93)	3(3.45)	9(10.34)
**Marwick, 2011**		Appropriate	57(33.53)	20(11.76)	10(5.88)	1(0.59)
		Under/Over	12(7.06)	36(21.18)	23(13.53)	11(6.47)
**Hashem, 2015**		Appropriate	8(4)	65(32.5)	14(7)	3(1.5)
		Under/Over	60(30)	37(18.5)	10(5)	3(1.5)
**Total **		Appropriate	122(17.09)	113(15.83)	36(5.04)	41(5.74)
		Under/Over	122(17.09)	175(24.51)	62(8.68)	43(6.02)

Data are presented as number (%) if they were available in the studies.

## 5. Limitations

There was a high amount of heterogeneity in some of our syntheses, but the small number of the included studies that had evaluated distinct types of study outcomes did not allow us to have a comprehensive review of the cause of the heterogeneity. Since all included studies had similar inclusion criteria for their patient recruitment, various factors could have affected the results, causing heterogeneity. One of these factors that our study shows to modify the heterogeneity between the studies is the different study settings in the included articles. Study ‎setting was emergency department (ED) in 4 studies; while 3 studies evaluated hospitalized patients (9-11).

## 6. Conclusion 

CREST classification demonstrates good precision in deciding the duration of hospitalization or observation at the ED; however, the reliability of this guideline in antimicrobial agent choice or route of antibiotic administration remains unclear; and using these classifications had not been able to prevent over/undertreatment with antibiotics, which might be due to inadequate and vague description and potentially overlapping definition of each class. ‎

## 7. Declarations

### 7.1. Acknowledgments

We would like to thank the Clinical Research Development Unit of Peymanieh Educational and Research and Therapeutic Center of Jahrom University of Medical Sciences for providing facilities for this work.

### 7.2. Funding

This research did not receive any grant from funding agencies in the public, commercial, or non-profit sectors.

### 7.3. Conflict of interest statement

The authors have declared that no competing interests exist.

### 7.4. Author contribution

HA, SH and SRH conceptualized the study questions and performed revisions. NK, NH, AM and SJ performed the searches. FM, MF and RA, BA, BSH conducted the statistical analyses. Other authors provided the draft of the manuscript.

### 7.5. Ethical Considerations

All ethical principles are considered in this article.
